# The Implementation of Precise Point Positioning (PPP): A Comprehensive Review

**DOI:** 10.3390/s23218874

**Published:** 2023-10-31

**Authors:** Mohamed Elsheikh, Umar Iqbal, Aboelmagd Noureldin, Michael Korenberg

**Affiliations:** 1 Electronics and Electrical Communication Engineering Department, Tanta University, Tanta 31512, Egypt; m1_essam@ieee.org; 2 Electrical and Computer Engineering Department, Queen’s University, Kingston, ON K7L 3N6, Canada or aboelmagd.noureldin@rmc.ca (A.N.); korenber@queensu.ca (M.K.); 3 Electrical and Computer Engineering Department, Mississippi State University, Starkville, MS 39762, USA; 4 Electrical and Computer Engineering Department, Royal Military College of Canada, Kingston, ON K7K 7B4, Canada

**Keywords:** GNSS, precise positioning, PPP, PPP errors, PPP corrections, IGS

## Abstract

High-precision positioning from Global Navigation Satellite Systems (GNSS) has garnered increased interest due to growing demand in various applications, like autonomous car navigation and precision agriculture. Precise Point Positioning (PPP) offers a distinct advantage over differential techniques by enabling precise position determination of a GNSS rover receiver through the use of external corrections sourced from either the Internet or dedicated correction satellites. However, PPP’s implementation has been challenging due to the need to mitigate numerous GNSS error sources, many of which are eliminated in differential techniques such as Real-Time Kinematics (RTK) or overlooked in Standard Point Positioning (SPP). This paper extensively reviews PPP’s error sources, such as ionospheric delays, tropospheric delays, satellite orbit and clock errors, phase and code biases, and site displacement effects. Additionally, this article examines various PPP models and correction sources that can be employed to address these errors. A detailed discussion is provided on implementing the standard dual-frequency (DF)-PPP to achieve centimeter- or millimeter-level positioning accuracy. This paper includes experimental examples of PPP implementation results using static data from the International GNSS Service (IGS) station network and a kinematic road test based on the actual trajectory to showcase DF-PPP development for practical applications. By providing a fusion of theoretical insights with practical demonstrations, this comprehensive review offers readers a pragmatic perspective on the evolving field of Precise Point Positioning.

## 1. Introduction

The Global Positioning System (GPS) was originally designed to achieve accuracy within a few meters, employing code measurements. At its inception, the designers had not foreseen the prospects for finer precision [1]. The use of GPS carrier-phase measurements to achieve centimeter-level accuracy was first explored by Counselman [2,3]. Because of their short wavelengths, these carrier-phase measurements offer higher precision than their code counterparts. For example, the carrier wavelengths for GPS L1 and L2 frequencies are about 19 cm and 24 cm, respectively. On the other hand, the duration of 1 bit in the GPS coarse/acquisition (C/A) code is 1μs, corresponding to about 300 m, while 1 bit in the P1 code, which is 10 times faster, corresponds to about 30 m [4].

The Integer Ambiguity (IA) is a paramount challenge for using precise GNSS carrier-phase measurements. The IA is the unknown initial number of full carrier cycles on the path from satellite to receiver. Achieving the desired precision is contingent on resolving this IA [1]. In a broader sense, Differential GPS (DGPS), or DGNSS in general, is engineered to bolster GNSS receiver performance, drawing upon data from one or more strategically positioned reference stations [5]. The core principle of DGNSS is that when the baseline length—defined as the distance between the rover receiver and the reference station—is adequately short (e.g., less than 40 km), certain errors, including atmospheric delays, satellite orbit, and satellite clock errors, become spatially correlated. These correlated errors can then be effectively eliminated using differencing techniques. The Real-Time Kinematics (RTK) is a carrier-phase-based DGNSS technology that provides centimeter-level real-time positioning accuracy [4]. Nonetheless, the overheads associated with reference station setup and the inherent baseline length constraints underscore the limitations of DGNSS.

Introduced in 1997, Precise Point Positioning (PPP) is a technique that facilitates the precise and accurate estimation of a user’s global position using a single GNSS receiver [6]. PPP determines the absolute receiver position, relying exclusively on the rover receiver’s GNSS measurements and a global spectrum of precision correction data. Since PPP must consider all error sources neglected in DGNSS, its pivotal challenge remains the convergence time required for resolving IA to achieve centimeter-level precision [7]. However, the ability of PPP to provide a globally applicable solution via a single receiver has gained significant interest within the research community, leading to ongoing efforts to overcome its limitations [8].

PPP can be applied in many aspects of our daily life. For example, in [9], PPP was integrated with high-end inertial sensors for lane-level autonomous land vehicle navigation on highways. Other papers investigated the integration of PPP with low-cost inertial sensors for land vehicle navigation in GNSS-challenging environments [10,11]. PPP was also applied for precision agriculture [12] and atmospheric monitoring [13]. In [14], PPP with the BeiDou B2b products was used for earthquake monitoring. Several open-source software tools can be used for PPP, such as RTKLIB [15], GAMP [16], PPPH [17], PPPLib [18], raPPPid [19], and PPP-ARISEN [20].

The main motivation behind this paper is to provide a basis of PPP understanding for young researchers and developers, so they can understand the concepts behind these open-source PPP tools and even go further to develop their own implementations. While this article draws from the first author’s PhD thesis on integrating PPP and inertial technologies [21], it is more to the point, emphasizing the granular implementation of standard PPP accompanied by hands-on examples. Additionally, it incorporates contemporary references of PPP applications, the latest developments in the field, and recent updates on PPP correction formats and data sources.

The rest of this paper is organized as follows. Section 2 delves into the PPP error sources and their mitigation strategies. Subsequently, Section 3 presents an overview of various PPP models, laying emphasis on the dual-frequency (DF) variants. Section 4 shares empirical results derived from a standard DF-PPP application using both static and kinematic data. Concluding the paper, Section 5 encapsulates the challenges linked with PPP and offers potential solutions.

## 2. Error Sources in PPP

For PPP to attain centimeter-level precision, it is imperative to eliminate or significantly mitigate range errors. Many of these errors, such as the Sagnac effect (attributable to Earth’s rotation), relativistic clock effects, satellite orbit and clock errors, and atmospheric delays, are also prevalent in Standard Point Positioning (SPP), leading to accuracies on the order of several meters [22]. The correction models employed for mitigating the Sagnac effect and relativistic clock errors in SPP equally apply to PPP; therefore, they are not elaborated on in this discussion.

Conversely, the broadcast ephemeris data employed in SPP to calculate the atmospheric delays and the errors in satellite position and clock are insufficient for reaching the targeted PPP accuracy. For instance, the GPS Klobuchar’s ionospheric model can correct around 50% rms of the ionospheric error [23]. Other broadcast ionospheric models also provide limited improvement to the positioning accuracy [24]. Therefore, more precise corrections and refined models are required in PPP. Furthermore, accounting for additional error sources, including antenna-induced discrepancies and site displacement errors, becomes crucial as we venture into centimeter-level precision.

### 2.1. Ionospheric Delays

The ionosphere is the ionized part of the atmosphere between 50 and 1000 km above sea level. Fluctuations in solar activity induce variations in the ionization levels, thereby impacting the refractive indices of the ionosphere’s various layers. This, in turn, alters the transit time of GNSS signals. Based on the solar activity’s intensity and the satellite’s elevation angle, ionospheric delays can induce ranging errors of up to 100 m [23]. It is essential to note that such delays are frequency-dependent. In the context of the standard DF-PPP, the DF measurements’ ionospheric-free (IF) combination is employed to counteract the first-order ionospheric error. The IF pseudorange $P_{IF}$ and carrier-phase $\Phi_{IF}$ measurements are articulated as follows:

(1) $$\begin{array}{cc} P_{IF} & =\alpha_{IF}P_{1}+\beta_{IF}P_{2}\\ \Phi_{IF} & =\alpha_{IF}\Phi_{1}+\beta_{IF}\Phi_{2} \end{array}$$

where $P_{i}$ and $\Phi_{i}$ represent the pseudorange and carrier-phase measurements of the GNSS frequency $f_{i}$, respectively. The IF coefficients are denoted as $\alpha_{IF}=f_{1}^{2}/\left(f_{1}^{2}-f_{2}^{2}\right)$ and $\beta_{IF}=-f_{2}^{2}/\left(f_{1}^{2}-f_{2}^{2}\right)$, where $f_{1},f_{2}$ correspond to the carrier frequencies of the combined signals.

The IF combination effectively eliminates up to 99.99% of the slant delay [25]. However, a trade-off exists: the resulting IF measurements exhibit increased noise levels compared with individual observations. For instance, the GPS IF observations are approximately threefold noisier than the uncombined L1 and L2 code and phase GPS observations [1]. Furthermore, higher-order ionospheric effects can introduce error magnitudes of up to 2 cm. These effects may also be modeled for applications requiring millimeter-level accuracy [7].

In the case of uncombined GNSS observations, the PPP algorithm can employ the Global Ionospheric Maps (GIMs) provided by the International GNSS Service (IGS) [26,27]. The IGS GIMs contain grid-based ionospheric Vertical Total Electron Content (VTEC) corrections, which can be interpolated and mapped to calculate the slant TEC (STEC) at the GNSS receiver location. The mapping is typically performed under the thin-layer assumption, which presumes that the ionosphere is just a thin layer at a fixed altitude between 350 and 450 km. The intersection between the ray from the satellite to the receiver and the thin layer is called the Ionosphere Pierce Point (IPP). The thin-layer assumption can introduce up to 10 m vertical range errors, reaching two or three folds at low elevations. More details about the calculation of the IPP and mapping process can be found in [28,29].

The IGS ionospheric products are available in a rapid product with a latency of less than 24 h and a final product with a latency of approximately 11 days. Both products can be used in postmission applications and are provided in the IONosphere Map EXchange (IONEX) format [30]. The Centre National d’Etudes Spatiales (CNES) was the first IGS analysis center to broadcast real-time ionospheric corrections [29]. The ionospheric delay *I*, in meters, is related to the STEC with the formula

(2) $$I=\frac{40.3\times 10^{16}}{f^{2}}\times STEC$$

where *f* is the signal frequency in Hz.

### 2.2. Tropospheric Delays

The troposphere, situated directly above Earth’s surface, predominantly comprises dry gases interspersed with water vapor. Distinct from the ionosphere’s dispersive nature, the troposphere is nondispersive concerning GNSS frequencies. However, its refractive properties decelerate GNSS signal propagation, subsequently resulting in a delay [5]. This tropospheric delay encompasses dry and wet constituents, with a total delay ranging between 2 and 25 m, depending on the satellite’s elevation angle and the atmospheric density profile. The dry segment contributes to approximately 90% of the tropospheric delay and is amenable to precise modeling.

Conversely, the wet component, constituting roughly 10% of the tropospheric delay, poses modeling challenges due to the localized variance in water vapor content [31]. The total tropospheric delay can be accurately modeled for SPP using models such as the Saastamoinen model [32]. Still, these models fall short of the meticulous precision requisites of PPP.

For precise positioning, each satellite’s total tropospheric delay *T* can be expressed as [7]

(3) $$T=M_{h}\left(E\right)Z_{h}+M_{w}\left(E\right)Z_{w}+M_{g}\left(E\right)\left(G_{N}\cos\left(Az\right)+G_{E}\sin\left(Az\right)\right)$$

where *E* and Az denote the satellite elevation and azimuth angles, respectively. The hydrostatic or dry component of the delay is characterized by the Zenith Hydrostatic Delay (ZHD), denoted as $Z_{h}$, scaled by an elevation-dependent mapping function $M_{h}$. Similarly, the Zenith Wet Delay (ZWD), denoted as $Z_{w}$, is scaled by its corresponding mapping function $M_{w}$. The third term in the equation accounts for the horizontal gradients in both north and east directions, scaled by a mapping function $M_{g}$. However, the impact of this term is generally on the order of millimeters and can be ignored in most applications.

The ZHD can be calculated using Saastamoinen’s formula [32] as given by [33]

(4) $$Z_{h}=\frac{0.0022768P}{1-0.00266\cos\left(2\varphi \right)-2.8\times 10^{-7}h}$$

where *P* represents the surface pressure expressed in hPa (One hPa (hectopascal) =100 Pa =1 millibar). The variable φ denotes the latitude, whereas *h* represents the height in meters. For achieving centimeter-level accuracy in PPP, the pressure (in hPa) can be approximated using Berg’s formula $P=1013.25{\left(1-2.2557\times 10^{-5}h\right)}^{5.2568}$ [34]. Conversely, the ZWD cannot be determined with the same level of precision. Therefore, the initial value of the ZWD is obtained from Saastamoinen’s model, and the residual error is treated as an unknown parameter and estimated by the navigation filter.

The mapping functions, $M_{h}$ and $M_{w}$, primarily depend on the satellite’s elevation angle. All existing mapping functions are derived from the continued fractions introduced by Marini [35] with normalization to unity at the zenith

(5) $$M\left(E\right)=\frac{1+\frac{a}{1+\frac{b}{1+c}}}{\sin E+\frac{a}{\sin E+\frac{b}{\sin E+c}}}$$

where *a*, *b*, and *c* are constant coefficients with small values of much less than 1. These coefficients vary for the dry and wet delay components. The Vienna Mapping Function 1 (VMF1) [36] is currently recognized as the most accurate mapping function for precise geodetic PPP solutions [37]; however, its application necessitates supplementary data from numerical weather models. On the other hand, Neill Mapping Functions (NMF) [38] rely solely on the receiver’s latitude, elevation, and the day of the year. Despite being less precise, the accuracy of NMF is adequate for various applications, such as vehicle navigation [9].

### 2.3. Satellite Orbit and Clock Errors

Knowing the precise satellite’s position in space is crucial for the estimation of the distance between the satellite and receiver, which is required to calculate the receiver’s position. In SPP, the information in the broadcast ephemeris is employed to determine the satellite position. However, the accuracy offered by broadcast ephemerides is suboptimal for high-precision applications, leading to a demand for more accurate ephemerides. Such precise satellite orbit data can be obtained from commercial providers or the IGS open-access products [26]. The IGS offers a spectrum of products with varying degrees of accuracy and latency. The final IGS products are the most accurate products, which feature an orbital accuracy of approximately 2.5 cm; nevertheless, they are available with a latency of 12–18 days [27].

Another potential source of error originates from satellite clock instabilities. The clock error model in the broadcast ephemeris also lacks the required accuracy for PPP, creating the need for more precise clocks. This high-accuracy orbital and clock data are conventionally stored in the Standard Product 3 (SP3) format, often with epoch intervals of 15 min or less. The latest version of SP3 at the time of this paper is SP3-d [39]. Both the orbital and clock data need to be interpolated to acquire exact positional and temporal details at the moment of signal transmission. Orbit interpolation is commonly achieved using a Lagrange polynomial of 9th or 10th order, yielding centimeter-level accuracy [40,41]. Linear interpolation, conversely, is generally sufficient for clock errors.

Although SP3 files do contain clock error data, higher-rate data are preferable due to the stochastic nature of clock errors in contrast to the relatively smooth satellite orbits. The IGS provides such high-rate clock data, typically at intervals of 5 min or 30 s, using the clock Receiver Independent Exchange (RINEX) format [42]. It is feasible to utilize SP3 data for the orbits and high-rate data for the clocks; however, it is necessary to maintain data consistency from the same analysis center.

Originally, IGS products were generated only for GPS and subsequently for GPS and GLONASS. The extension to support all GNSS constellations was realized through the IGS Multi-GNSS Experiment (MGEX) project [43,44]. Seven IGS analysis centers now provide orbits and clock products for Multi-GNSS, namely, Centre National d’Etudes Spatiales (CNES), Center for Orbit Determination in Europe (CODE), GeoForschungsZentrum Potsdam (GFZ), Information and Analysis Center (IAC), Shanghai Observatory (SHAO), Wuhan University, and the Japan Aerospace Exploration Agency (JAXA).

To meet the growing demand for real-time PPP, the IGS officially launched its Real-Time Service (IGS RTS) on 1 April 2013 [45]. The real-time corrections are disseminated to the users via the Internet using protocols such as NTRIP (Networked Transport of Radio Technical Commission for Maritime Services (RTCM) via Internet Protocol) [26]. RTS messages conform to the RTCM State-Space Representation (SSR) format. The SSR parameters are expressed as relative corrections to the broadcast ephemeris-derived values rather than as absolute precise satellite orbits and clocks to minimize the transmission bandwidth [45].

### 2.4. Code Biases

Code biases, originating from time delays induced by satellite and receiver hardware, are influenced by various factors, including signal frequency, waveform, and tracking technology [46,47]. Receiver code biases are typically assumed to be independent of the satellite for a given code observation and are incorporated in the estimated receiver clock bias [7,47]. In contrast, satellite code biases are usually represented in a differential form rather than their absolute values.

Differential Code Bias (DCB) denotes the time delay between two code observations of the same satellite at the same or different frequency [48]. For instance, a specific DCB exists between the P1 code and the C/A code, hereafter referred to as C1, both operating at the L1 frequency of GPS. This DCB is symbolized as $DCB_{P1-C1}$. Likewise, $DCB_{P1-P2}$ represents the DCB between P1 and P2 code observations, which function at different frequencies.

For GPS and GLONASS systems, satellite clock corrections in both broadcast and precise ephemerides are calculated based on the IF combination of P-code observations at L1 and L2 frequencies (P1/P2) [7]. Consequently, there is no necessity for DCB corrections in DF-PPP when employing the P1/P2 IF combination. However, when alternative code combinations are utilized instead of P1/P2, the appropriate DCB correction must be applied. For example, when using the IF combination C1/P2, the term $c\alpha_{IF}DCB_{P1-C1}$ is introduced to adjust the IF code observation, where *c* is the speed of light and $\alpha_{IF}$ is as defined in Section 2.1. This principle also applies to other constellations and frequencies.

**Figure 1:**
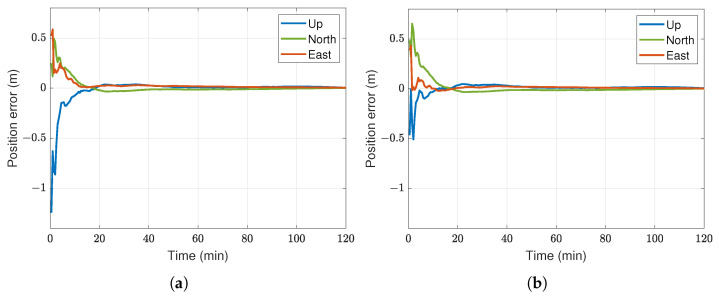
Impact of DCB corrections on PPP convergence utilizing two hours of static data from the YELL station on 20 July 2023: (**a**) without DCB corrections, (**b**) $DCB_{P1-C1}$ corrections applied.

Although the high precision of PPP is predominantly derived from carrier-phase observation, incorporating DCBs reduces convergence time and expedites ambiguity resolution [7]. Figure 1 illustrates the impact of DCB on float-ambiguity DF-PPP convergence, utilizing two hours of static GPS data from the YELL IGS station. The final IGS products were adopted in this example, and all other centimeter-level PPP errors were accounted for. The application of $DCB_{P1-C1}$ yielded lower initial solution errors and accelerated convergence, especially in eastward and upward directions for this specific dataset. After the convergence, the influence of the carrier phase becomes dominant, and the effect of the DCB corrections is no longer significant, as evidenced by the nearly identical trajectories in the corresponding graphs.

Applying $DCB_{P1-P2}$ in single-frequency PPP is indispensable when incorporating P1 or P2 observations. Additional DCB parameters may be invoked should alternative code observations be considered. In the case of GPS, the $DCB_{P1-P2}$ value is available through the Timing Group Delay (TGD) parameter in the broadcast navigation message [49]. The new civil navigation message, started with Block IIR-M of the GPS satellites, contains Inter-Signal Correction (ISC) parameters, which provide the DCBs between the L1 P(Y) or P1 code and L1 C/A (C1) and between L1 P(Y) and L2C code. Alternatively, the DCBs for different frequencies and constellations can be obtained post mission through the IGS products [50] or in real time through the IGS RTS.

The DCB terms are often employed with the satellite clock correction $dt_{P_{IF}}^{s}$, which is referenced to the P1/P2 IF combination $P_{IF}$ for GPS and GLONASS systems. In the case of the single-frequency GPS C1 observation, the satellite clock error $dt_{C1}^{s}$ can be expressed as [47,49]

(6) $$\begin{array}{cc} dt_{C1}^{s} & =dt_{P_{IF}}^{s}-TGD+ISC_{L1C/A}\\ & =dt_{P_{IF}}^{s}+\frac{f_{L2}^{2}}{f_{L1}^{2}-f_{L2}^{2}}DCB_{P1-P2}+DCB_{P1-C1} \end{array}$$

Equation (6) relates the GPS broadcast parameters (TGD, ISC) and the DCBs as defined by the IGS. The bias parameters are expressed in units of seconds, and $f_{L1}$, $f_{L2}$ represent the carrier frequencies of the L1 and L2 GPS bands. Although there may be an arbitrary offset between the broadcast and IGS parameters, it should not impact the final positioning solution as it is absorbed into the receiver clock error. It is also worth mentioning that the DCB files currently adopt the latest RINEX observation code convention [51], e.g., C1C/C1W/C2W for GPS C1/P1/P2 codes, respectively.

Each GNSS employs its timing system in multiconstellation PPP, introducing bias components that represent the delays between the systems. These biases are characterized by the Inter-System Bias (ISB) and are typically estimated for each constellation as a time offset relative to GPS [52,53].

The Inter-Frequency Bias (IFB) is a code bias that exists in GLONASS legacy signals. The GLONASS legacy signals adopt the Frequency Division Multiple Access (FDMA) technique, whereby each satellite transmits signals at a slightly varying frequency from the other satellites. In contrast, other GNSSs are not affected by these biases, since they operate on consistent frequencies using the Code Division Multiple Access (CDMA) technique. These IFBs in GLONASS are satellite-dependent receiver biases that are not easily isolable or absorbed into other parameters. Therefore, because code biases influence the initial PPP convergence, GLONASS IFBs are generally neglected [46].

### 2.5. Phase Biases

Hardware delays also influence carrier-phase observations. However, their impact varies compared with that of code observations. When ambiguities are treated as float values, phase biases from the same constellation are absorbed into the ambiguity terms. Thus, in float-ambiguity PPP, phase biases typically raise no concerns except for the ISB terms, which must be incorporated into the phase observation in Multi-GNSS scenarios. It is important to note that while the ISB value for phase observation might differ from that of code ISB, any discrepancy is lumped into the float ambiguities.

In contrast, in ambiguity-fixed PPP, it is crucial to separate the phase biases to retrieve the integer nature of ambiguities. While this article focuses on the standard float PPP, an overview of ambiguity-fixed PPP can be found in Section 3.3.

### 2.6. Antenna Phase Center Effects

The distance between a GNSS satellite and a receiver is measured between their respective Antenna Phase Centers (APCs). If the precise satellite orbit data are referred to the satellite Center of Mass (CoM), such as in the IGS SP3 files and some real-time products, the difference between the satellite CoM and APC must be accounted for. On the other hand, the receiver’s reference location is often determined at a marker position that can be the Antenna Reference Point (ARP) or another point on the ground below the receiver antenna. Consequently, the difference between the receiver reference point and its APC must be considered.

APC errors are frequency-dependent and can be classified into Phase Center Offset (PCO) and Phase Center Variation (PCV). Calibrated corrections for these errors are available in the ANTenna Exchange (ANTEX) format file provided by the IGS [54]. The calibration files for the antenna phase center can be accessed through the IGS online archive [55]. Starting from GPS week 2238, 27 November 2022, the IGS adopted the IGS20 reference frame and igs20.atx antenna calibration files [56].

#### 2.6.1. Satellite APC Errors

The PCO of a satellite is defined as the distance between its CoM and the mean APC, expressed in the satellite body-fixed frame. The IGS definition for this frame is that the coordinate system’s origin is at the satellite’s CoM, the z-axis is parallel to the antenna boresight, the y-axis is aligned with the rotation axis of the solar panels, and the x-axis completes the right-handed system [57].

**Figure 2:**
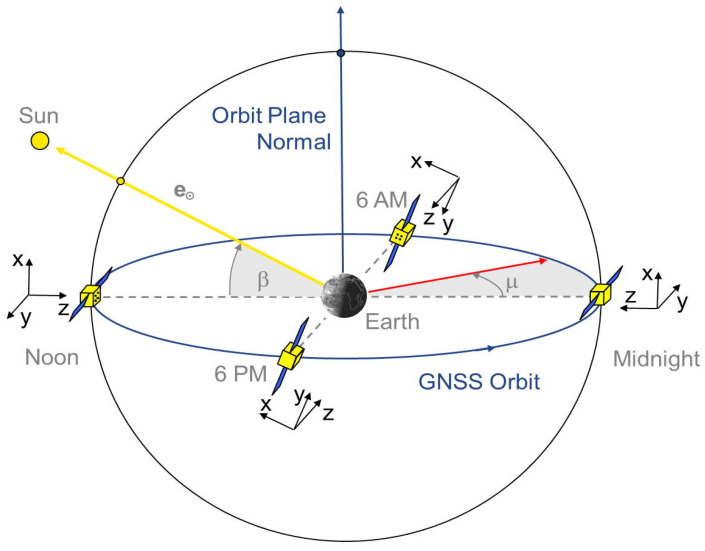
Yaw-steering mode of GNSS satellite orientation (after [57]).

During nominal operation conditions, GNSS satellites employ the “yaw-steering” attitude control mode, which necessitates continuous rotation around the z- and y-axes, as shown in Figure 2. Alternative attitude control modes are deployed during eclipse periods or for low angular values of β (defined in Figure 2); nonetheless, these modes are beyond the scope of this study. The unit vectors of the satellite body frame $\left({\hat{x}}_{s},{\hat{y}}_{s},{\hat{z}}_{s}\right)$ in the yaw-steering mode can be expressed in the Earth-Centered Earth-Fixed (ECEF) as follows [41,57]

(7) $$\begin{array}{cc} {\hat{z}}_{s} & =-\frac{r_{s_{\mathrm{CoM}}}}{∥r_{s_{\mathrm{CoM}}}∥} \end{array}$$

(8) $$\begin{array}{cc} {\hat{y}}_{s} & =\frac{{\hat{z}}_{s}\times e_{s}}{∥{\hat{z}}_{s}\times e_{s}∥} \end{array}$$

(9) $$\begin{array}{cc} {\hat{x}}_{s} & ={\hat{y}}_{s}\times {\hat{z}}_{s} \end{array}$$

where $e_{s}=\frac{R_{☼}-r_{s_{\mathrm{CoM}}}}{∥R_{☼}-r_{s_{\mathrm{CoM}}}∥}$, $r_{s_{\mathrm{CoM}}}$ is the CoM satellite position vector, and $R_{☼}$ represents the sun’s position vector, both in the ECEF frame. The sun’s position can be calculated from the planetary ephemerides [58] or simply from analytical formulas [59].

If the satellite position is expressed in the ECEF frame, the PCO corrections must also be transformed to the ECEF frame and subsequently added to the satellite position as follows:

(10) $$\begin{array}{cc} {\mathrm{PCO}}_{\mathrm{ECEF}}^{s} & =\left[\begin{array}{ccc} {\hat{x}}_{s} & {\hat{y}}_{s} & {\hat{z}}_{s} \end{array}\right]{\mathrm{PCO}}^{s} \end{array}$$

(11) $$\begin{array}{cc} r_{s} & =r_{s_{\mathrm{CoM}}}+{\mathrm{PCO}}_{\mathrm{ECEF}}^{s} \end{array}$$

where ${\mathrm{PCO}}^{s}$ is the satellite PCO vector in the satellite body frame (from the ANTEX file), ${\mathrm{PCO}}_{\mathrm{ECEF}}^{s}$ contains the offset values in the ECEF frame, and $r_{s}$ represents the corrected satellite position vector at the APC. The magnitude of the satellite offset vector varies between 0.5 and 3 m [7], which may induce centimeter-level positioning errors.

The satellite PCV refers to the change in the APC as a function of the satellite nadir angle. IGS provides calibrated satellite PCV values in its ANTEX file for specific nadir angle ranges, up to $17^{\circ }$ for GPS and up to $15^{\circ }$ for GLONASS with steps of $1^{\circ }$. For Galileo, the range is up to $20^{\circ }$ with a step of $0.5^{\circ }$, while there are no PCVs for BDS satellites so far. The relevant PCV values are obtained through linear interpolation at the calculated satellite nadir angle. The nadir angle θ is defined as the angle between the direction from the satellite toward the center of the earth and the Line-of-Sight (LOS) vector between the satellite and receiver. It can be calculated as

(12) $$\theta =\arccos\left(-u_{r}^{s}⊙-{\hat{z}}_{s}\right)=\arccos\left(u_{r}^{s}⊙{\hat{z}}_{s}\right)$$

where $u_{r}^{s}$ is the LOS unit vector from the receiver to the satellite in the ECEF frame and ⊙ is the dot-product operator.

After interpolation, the derived PCV values are subtracted from both the code and carrier-phase observations. Nonetheless, the satellite PCVs are typically within the millimeter range and, thus, influence only those applications demanding millimeter-level accuracy.

#### 2.6.2. Receiver APC Errors

**Figure 3:**
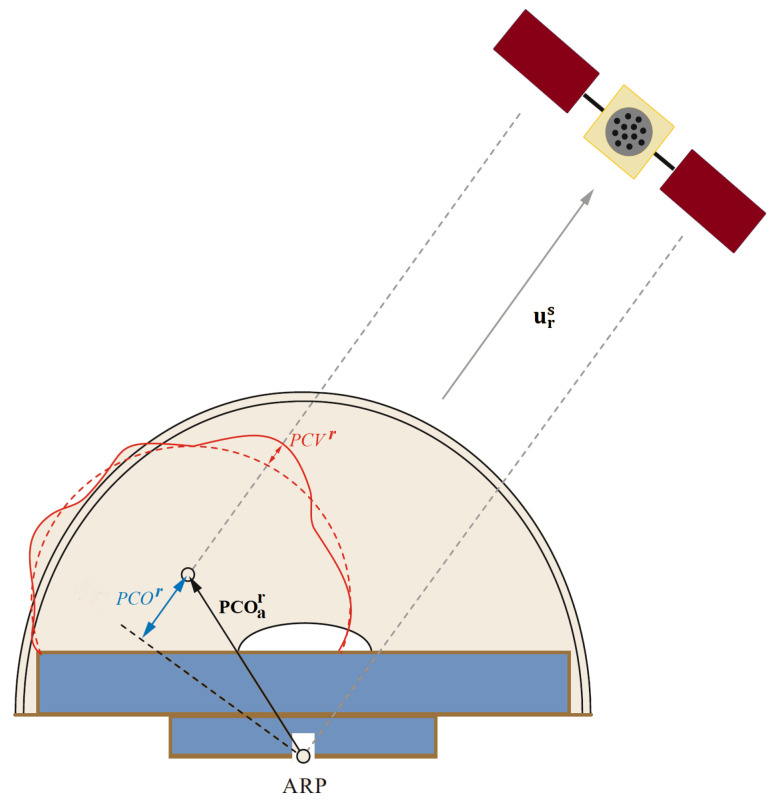
Illustration of the receiver antenna phase center errors: phase center offset ($PCO^{r}$) and phase center variation ($PCV^{r}$) (after [60]).

The antenna phase center is inherently nonphysical and exhibits frequency dependence. As a result, the location of a receiver antenna typically references the ARP. As the IGS delineates, the ARP is where the antenna’s base intersects its vertical axis of symmetry [40]. The receiver PCO represents the distance between the average APC and the ARP. Antenna manufacturers typically provide these PCO values, or at least the vertical PCO component, for each carrier frequency. Furthermore, the IGS ANTEX file includes calibrated PCO values for numerous recognized antenna models. The effective receiver PCO value ($PCO^{r}$) signifies the projection of the PCO vector within the antenna-fixed frame, ${\mathrm{PCO}}_{a}^{r}$, on the negative LOS vector connecting the receiver and satellite antennas, as depicted in Figure 3 [60].

(13) $$PCO^{r}=-u_{r}^{s}\cdot \left(R_{a}^{e}\;{\mathrm{PCO}}_{a}^{r}\right)$$

where the matrix $R_{a}^{e}$ is the transformation matrix from the antenna-fixed frame to the ECEF frame.

The IGS ANTEX format specifies the receiver PCO values in the Local-Level Frame (LLF), where the offsets are expressed in the north, east, and up directions. However, when applying the rotation, care must be taken in ordering the axes. Assuming that the ${\mathrm{PCO}}_{a}^{r}$ vector is arranged such that its components are east (E), north (N), and up (U), respectively. Then, the rotation matrix $R_{a}^{e}$ is equal to $R_{l}^{e}$, which is the rotation from the LLF to the ECEF frame [61]

(14) Rae=Rle=−sin(λ)−sin(φ)cos(λ)cos(φ)cos(λ)−cos(λ)−sin(φ)sin(λ)cos(φ)sin(λ)−0−cos(φ)sin(φ)

where φ and λ are the latitude and longitude of the receiver, respectively.

The receiver PCV is depicted as a function of the satellite elevation and azimuth angles, as represented by the solid red curve in Figure 3. Calibrated PCV values for various antenna models can be extracted from the IGS ANTEX file. These values are computed at zenith angles ($zenith\;angle=90^{\circ }-elevation\;angle$) from 0 to $90^{\circ }$ in $5^{\circ }$ increments and are available in both azimuth-dependent and azimuth-independent formats. The azimuth-dependent values cover the 360-degree azimuth range in $5^{\circ }$ steps. Interpolation methods are employed to obtain PCV values at specific elevation and azimuth angles.

The receiver APC errors are systematic errors in the GNSS observations with magnitudes ranging between 5 to 15 cm for the PCO and up to 3 cm for the PCV [7]. The receiver PCO error primarily influences the estimated height component. It is important to note that the receiver’s APC corrections are often referenced to the ARP. If the target location is at a monument marker or geodetic point, an additional offset to the marker must be incorporated into ${\mathrm{PCO}}_{a}^{r}$ in (13). Moreover, consistency must be preserved when comparing the PPP solution with a reference solution, ensuring that receiver APC corrections are uniformly applied or omitted across both solutions.

The total receiver APC error can be written as

(15) $$APC_{r}=PCO_{r}+PCV_{r}$$

This error is subtracted from both code and phase measurements to compensate for the receiver APC effects.

### 2.7. Phase Wind-Up

GNSS satellites transmit primarily Right-Hand Circularly Polarized (RHCP) electromagnetic waves. This particular polarization aids in diminishing multipath effects and liberates the system from constraints concerning the relative orientation of the GNSS satellite and receiver antennas [7]. However, any deviation in the antenna’s orientation introduces a variation in the carrier-phase measurements, a phenomenon termed “phase wind-up” [62]. Notably, while code measurements remain unaffected by this phenomenon, a complete rotation of the receiving or transmitting antennas alters the measured carrier phase by a full cycle across all frequencies.

Although the GNSS signal includes both RHCP and left-hand components, for receivers situated at small angles from the satellite antenna boresight, the left-hand component can be disregarded [62]. Therefore, under the pure RHCP assumption, we can model the phase wind-up effect using the effective dipole method [62]. In this method, both the satellite and receiver antennas are represented as crossed dipole antennas. Assuming that $\hat{k}=-u_{r}^{s}$ is the unit vector from the satellite to receiver antennas, the effective dipole vectors for the satellite $D_{s}$ and receiver $D_{r}$ can be expressed as follows:

(16) $$\begin{array}{cc} D_{s} & ={\hat{x}}_{s}-\hat{k}\left(\hat{k}⊙{\hat{x}}_{s}\right)+\hat{k}\times {\hat{y}}_{s} \end{array}$$

(17) $$\begin{array}{cc} D_{r} & ={\hat{x}}_{r}-\hat{k}\left(\hat{k}⊙{\hat{x}}_{r}\right)+\hat{k}\times {\hat{y}}_{r} \end{array}$$

where ${\hat{x}}_{s}$ and ${\hat{y}}_{s}$ are the satellite dipole unit vectors, whereas the receiver dipole unit vectors are denoted as ${\hat{x}}_{r}$ and ${\hat{y}}_{r}$. These dipole vectors are deliberately selected to be perpendicular to the direction of wave propagation, adhering to the right-hand rule. As a result, the receiver dipole vectors (${\hat{x}}_{r}$, ${\hat{y}}_{r}$) typically align with the receiver unit vectors in either (east, north) or (north, west) directions. The unit vectors in the east and north directions can be derived from the first and second columns of the direction cosine matrix described in (14). The satellite dipole unit vectors are defined in (8) and (9).

The fractional component of the phase wind-up correction, denoted as $\Delta \phi_{w}$ in radians, can be written as

(18) $$\begin{array}{cc} \Delta \phi_{w} & =\mathrm{sign}\left(\zeta \right)\arccos\left(\frac{D_{s}⊙D_{r}}{∥D_{s}∥∥D_{r}∥}\right) \end{array}$$

(19) $$\begin{array}{cc} \zeta & =\hat{k}⊙\left(D_{s}\times D_{r}\right) \end{array}$$

To maintain the continuity of the phase wind-up correction, the integer part *N* is determined using the preceding correction value $\phi_{w_{prev}}$ as follows:

(20) $$N=\mathrm{nint}\left(\frac{\phi_{w_{prev}}-\Delta \phi_{w}}{2\pi }\right)$$

where nint is the nearest integer operator. The value of *N* can be initialized with zero. The total phase wind-up correction becomes

(21) $$\phi_{w}=2\pi N+\Delta \phi_{w}$$

Finally, the phase wind-up correction is subtracted from the carrier-phase observation. However, it is essential to ensure unit consistency throughout the calculations. Given that the correction in (21) is expressed in radians, it must be divided by 2π to convert it to cycles. Furthermore, if the carrier-phase observation is originally expressed in meters, the phase wind-up correction needs to be multiplied by the carrier wavelength.

### 2.8. Site Displacement Effects

PPP offers an absolute global solution in alignment with global terrestrial frames, such as the International Terrestrial Reference Frame (ITRF). The earth’s crust experiences displacements due to various natural phenomena, such as solid Earth tides, polar tides, ocean loading, atmospheric pressure loading, and accumulations of groundwater and snow. While such displacements might be deemed negligible in relative positioning over concise baselines, their implications are significant for an ITRF-compatible PPP solution [63].

This section addresses primary displacement effects, particularly those exceeding 1 cm, such as solid Earth tides, polar tides, and ocean loading. The secondary effects can be safely ignored for centimeter-level positioning. The vector Δr denotes the total site displacement in the ECEF frame. This vector must be added to the calculated receiver position vector $r_{r}$ to calculate its position in the ITRF $r_{r_{\mathrm{ITRF}}}$ as follows:

(22) $$\begin{array}{cc} r_{r_{\mathrm{ITRF}}} & =r_{r}+\Delta r\\ & =r_{r}+\left(\Delta r_{\mathrm{solid}}+\Delta r_{\mathrm{polar}}+\Delta r_{\mathrm{ocean}}\right) \end{array}$$

The following subsections describe the details of each displacement component in (22).

#### 2.8.1. Solid Earth Tides

The gravitational forces exerted by the sun and the moon, responsible for ocean tides, also influence the solid Earth, leading to both horizontal and vertical site displacements. These displacements are quantified through a spherical harmonics expansion characterized by Love and Shida numbers [63]. To achieve an accuracy level of 5 mm, it is adequate to consider only the second-degree tides and a height correction factor [64]. Following this approximation, the displacement vector $\Delta r_{\mathrm{solid}}$ for a station at position $r_{r}$ can be expressed in the ECEF frame as

(23) $$\begin{array}{c} \Delta r_{\mathrm{solid}}=\sum_{j=☼,☾}\frac{GM_{j}}{GM}\frac{{∥r_{r}∥}^{4}}{{∥R_{j}∥}^{3}}\left\{\left[3l_{2}\left(\hat{R_{j}}\cdot \hat{r_{r}}\right)\right]\hat{R_{j}}+\left[3\left(\frac{h_{2}}{2}-l_{2}\right){\left(\hat{R_{j}}\cdot \hat{r_{r}}\right)}^{2}-\frac{h_{2}}{2}\right]\hat{r_{r}}\right\}\\ +\left[-0.025\sin\left(\varphi \right)\cos\left(\varphi \right)\sin\left(\theta_{g}+\lambda \right)\hat{r_{r}}\right] \end{array}$$

where GM is the earth’s gravitational parameter and $\hat{r_{r}}$ denotes the geocentric unit vector in the direction of $r_{r}$, while $GM_{j}$ represents the gravitational parameter and $R_{j}$ represents the geocentric position with the corresponding unit vector $\hat{R_{j}}$ for the sun when j=☼ and the moon when j=☾. The nominal second-degree Love and Shida numbers $\left(l_{2},h_{2}\right)$ can be approximated as (0.609,0.085).

The height correction, the last term in (23), depends on the station latitude φ, longitude λ, and the Greenwich mean sidereal time $\theta_{g}$. More harmonics and more accurate Love and Shida number values may be considered for high-precision applications [65]. As elaborated in Section 2.6.1, the sun and moon positions can be calculated from the planetary ephemerides or analytical formulas.

The magnitude of the displacement vector in (23) can reach 30 cm and 5 cm in the vertical and horizontal directions, respectively. This vector consists of a permanent component at the decimeter level, which is latitude-dependent, and a periodic component [7,63]. While the periodic component may be averaged out over long static datasets, it remains crucial to account for solid Earth tides due to the permanent component.

#### 2.8.2. Polar Tides

The polar tide, also known as polar motion, represents the fluctuation in the earth’s rotation axis’s geocentric position relative to the earth’s crust. The consequent disturbances to the centrifugal force linked with the earth’s daily rotation deforms the earth [66]. Displacements arising from polar tides, in mm, calculated in east, north, and up directions, are given by [65]

(24) Δre=−9sinφm1sinλ−m2cosλΔrn=−9cos2φm1cosλ+m2sinλΔru=−33sin2φm1cosλ+m2sinλ

where $m_{1}=\left(x_{p}-\bar{x_{p}}\right)$ and $m_{2}=-\left(y_{p}-\bar{y_{p}}\right)$, both expressed in arc seconds (1 arc second = 1/3600 degree), represent the variations to the coordinates of the earth’s mean pole in the terrestrial reference frame. These variations are determined as the difference of the polar motion variables $\left(x_{p},y_{p}\right)$, which can be obtained from the IGS Earth rotation parameters product, and the mean pole $\left(\bar{x_{p}},\bar{y_{p}}\right)$ from the International Earth Rotation and Reference Systems Service (IERS) model defined in Equation (7.25) in [65].

The maximum magnitude of the polar tide displacement is 25 mm and about 7 mm in the vertical and horizontal directions, respectively [65]. The corresponding displacement in the ECEF frame can be calculated using the rotation matrix in (14)

(25) $$\Delta r_{\mathrm{polar}}=R_{l}^{e}{\left[\begin{array}{ccc} \Delta r_{e} & \Delta r_{n} & \Delta r_{u} \end{array}\right]}^{T}$$

#### 2.8.3. Ocean Loading

Oceanic loading refers to the deformation of the earth’s crust caused by ocean tides. Analogous to solid tides, this effect has semidiurnal and diurnal cycles but with no permanent component. Typically, the induced site displacement is on the order of centimeters, predominantly in the vertical plane. However, in coastal regions, these displacements can escalate up to 10 cm [7]. The influence of oceanic loading may often be disregarded for daily static positioning or stations significantly inland. Conversely, it becomes imperative to account for this effect during shorter processing intervals, especially when in close proximity to oceanic bodies and when precision at the centimeter level is required in kinematic processing.

Ocean loading is characterized by the summation of eleven primary tidal harmonics; each harmonic *j* is characterized by its amplitude $A_{cj}$, phase $\phi_{cj}$, and time-varying argument $\chi_{j}\left(t\right)$ [65]. The amplitude and phase values of each depend on site and chosen model. These values can be obtained from the ocean loading service website [67], where they are represented in the south, west, and up directions. For each direction, the displacement Δc due to ocean loading can be calculated as

(26) $$\Delta c=\sum_{j=1}^{11}A_{cj}\cos\left(\chi_{j}\left(t\right)-\phi_{cj}\right)$$

The astronomical arguments $\chi_{j}\left(t\right)$ are calculated following the FORTRAN subroutines provided by IERS (https://iers-conventions.obspm.fr/content/chapter7/software/, accessed on 6 August 2023). The eleven harmonics in (26) encompass the semidiurnal $\left(M_{2},S_{2},N_{2},K_{2}\right)$, diurnal $\left(K_{1},O_{1},P_{1},Q_{1}\right)$, and long-period $\left(M_{f},M_{m},S_{sa}\right)$ tide waves.

The ocean loading displacement vector in the ECEF frame, denoted as $\Delta r_{\mathrm{ocean}}$, can be determined from the calculated Δc components similar to (25). However, before applying the same rotation matrix, the west and south ocean components must be converted to the east and north directions through sign inversion.

Finally, it is important to note that two key decisions should be made when downloading the amplitude and phase data from the ocean loading provider. The first choice pertains to the ocean tide model. As recommended by [65], the latest models should be used unless consistency with old models is necessary. The ocean loading service website [67] provides details on all available models. The Finite Element Solution (FES) model is an example of these models, and the latest version currently available is FES2014b. The second choice relates to whether CoM correction is required in the ocean model. While ocean tides influence the earth’s CoM, this correction is not required in PPP, as the IGS products are referenced to the ITRF, which is crust-fixed and remains unaltered by the earth’s CoM [7].

## 3. PPP Models

PPP exhibits a range of implementation modes contingent upon various determinants, including accessible constellations, management of the ionospheric delays, the decision to fix phase ambiguities, and the number of operational GNSS frequencies.

While ionospheric delays are frequently mitigated using IF measurement combinations, leveraging ionospheric corrections sourced from either local or global networks can expedite the convergence of the solution. Concerning ambiguity resolution (AR), the ambiguities might be estimated as float values or as fixed to their integer counterparts. Float PPP remains a prevalent choice, especially for real-time applications, due to the complexity of fixing integer ambiguities within the PPP context. Nevertheless, ambiguity-fixed PPP, or PPP-AR, has the advantages of shorter initial convergence time and improved accuracy, especially in the east direction [7,68]. PPP-RTK is a state-of-the-art technique that combines the complementary characteristics of PPP and RTK to achieve centimeter-level positioning in a short time [69].

Classified by the number of employed GNSS frequencies, implementations encompass single-frequency (SF), dual-frequency, and triple-frequency (TF) PPP. SF-PPP utilizes observations from a single GNSS frequency, which is useful when using low-cost SF GNSS receivers. SF-PPP has the advantages of low-cost hardware and shorter convergence time but to a limited accuracy range of the decimeter to submeter level, especially in the kinematic mode [70,71]. Recently, the availability of DF observations in low-cost GNSS receivers, even smartphones, has grown, reducing the interest in SF-PPP. In TF-PPP, observations from three GNSS frequencies are employed, e.g., GPS L1, L2, and L5. The third frequency’s main contribution is to shorten the convergence time and allow faster ambiguity resolution. Because of this, positioning accuracy can be improved in the short term due to faster convergence, but the contribution to positioning accuracy after convergence will be limited [72]. One limitation of the TF-PPP is that it is still not supported by all GNSS receivers in the market, especially low-cost ones [10].

The focus of this review is the implementation of DF-PPP. This section starts with a description of the GNSS code and carrier-phase observations and some of their valuable linear combinations. Then, these observations, in addition to the error sources correction and modeling discussed in Section 2, are used to build the standard DF-PPP and the ambiguity-fixed DF-PPP models. Finally, the PPP-RTK model is introduced.

### 3.1. GNSS Code and Phase Observations

The definition of GNSS pseudorange and carrier-phase observations is the fundamental component of all GNSS models. While the existing literature frequently relies on simplified equations to describe these observations, more detailed formulas are required to understand the impact of the various error sources detailed in Section 2 on GNSS observations. Hence, the pseudorange *P* and carrier-phase Φ observations from a specific GNSS satellite, both expressed in meters, can be represented as follows:

(27) $$\begin{array}{cc} P_{i} & =\rho^{\prime }+c\left(dt^{r}-dt^{s}\right)+b_{P_{i}}^{r}-b_{P_{i}}^{s}+T+I_{i}-v+PCV_{i}^{s}+APC_{i}^{r}+\epsilon_{P_{i}} \end{array}$$

(28) $$\begin{array}{cc} \Phi_{i} & =\rho^{\prime }+c\left(dt^{r}-dt^{s}\right)+b_{\Phi_{i}}^{r}-b_{\Phi_{i}}^{s}+T-I_{i}-v+PCV_{i}^{s}+APC_{i}^{r}+\lambda_{i}N_{i}+\frac{\lambda_{i}}{2\pi }\phi_{w}+\epsilon_{\Phi_{i}} \end{array}$$

In the above equations, the subscript *i* denotes the frequency index, *c* represents the speed of light, and $dt^{r}$, $dt^{s}$ stand for the receiver and satellite clock errors in seconds, respectively, while $b_{P_{i}}^{r},b_{P_{i}}^{s}$ are the receiver and satellite code biases and $b_{\Phi_{i}}^{r},b_{\Phi_{i}}^{s}$ are the receiver and satellite phase biases. *T* denotes the tropospheric delay; *I* represents the ionospheric delay; *v* accounts for the relativistic effects; $PCV_{i}^{s}$ signifies the satellite PCV error; $APC_{i}^{r}$ represents the receiver antenna phase center errors; and $\epsilon_{P_{i}}$, $\epsilon_{\Phi_{i}}$ denote the multipath and receiver noise for code and carrier phase, respectively, all expressed in meters. The integer ambiguity in cycles is symbolized as $N_{i}$, and the phase wind-up effect $\phi_{w}$ is measured in radians, as indicated in (21). Each term’s sign conforms to the correction models and formulas prevalent in the literature and in Section 2.

Furthermore, the symbol $\rho^{\prime }$ in (27) and (28) represents the geometric distance in meters between the receiver and satellite. The prime superscript signifies that this distance is contaminated by influences such as the Sagnac effect, satellite orbit errors, and site displacements. It also encompasses the satellite phase center offset if the satellite position or its corrections are referenced to the satellite CoM rather than APC.

In addition to individual GNSS observations, GNSS algorithms frequently employ various useful code and phase linear combinations. These linear combinations, for the case of two different frequencies, $f_{1}$ and $f_{2}$, generally take the following form [73]:

(29) $$\begin{array}{cc} P_{comb} & =\alpha P_{1}+\beta P_{2} \end{array}$$

(30) $$\begin{array}{cc} \Phi_{comb} & =\alpha \Phi_{1}+\beta \Phi_{2} \end{array}$$

where the subscript comb designates linear combination, while α,β signify the combination coefficients. The factor $\sqrt{\alpha^{2}+\beta^{2}}$ represents the extent to which the combined observation noise increases or decreases, assuming equal variance and uncorrelated noise from the two combined observations. Subsequently, this section briefly outlines some of these combinations.

#### 3.1.1. Ionospheric-Free (IF) Code and Phase

As previously introduced in Section 2.1 and represented by (1), the IF combination offers a solution to eliminate first-order ionospheric delays at the cost of increased noise levels.

#### 3.1.2. Geometric-Free (GF) Code and Phase

The GF combination is constructed by taking the difference between the observations of two frequencies, where $\alpha_{GF}=1$ and $\beta_{GF}=-1$. This approach effectively removes the geometric component and frequency-independent terms, leaving behind frequency-dependent terms, such as ionospheric delays and signal biases. GF combinations are useful in cycle slip detection and ionospheric total electronic content estimation.

#### 3.1.3. Wide-Lane (WL) Phase Observation

Another useful combination is the wide-lane phase $\Phi_{WL}$, derived by setting $\alpha_{WL}=f_{1}/\left(f_{1}-f_{2}\right)$ and $\beta_{WL}=-f_{2}/\left(f_{1}-f_{2}\right)$ in (30). $\Phi_{WL}$ introduces a new WL ambiguity $N_{WL}=N_{1}-N_{2}$ corresponding to a wavelength $\lambda_{WL}=c/\left(f_{1}-f_{2}\right)$. The WL wavelength exceeds L1 and L2 wavelengths, e.g., $\lambda_{WL}=86.2$ cm for GPS. The WL phase proves valuable in cycle slip detection and integer ambiguity resolution.

#### 3.1.4. Narrow-Lane (NL) Code Observation

The narrow-lane code $P_{NL}$ can be derived by setting $\alpha_{NL}=f_{1}/\left(f_{1}+f_{2}\right)$ and $\beta_{NL}=f_{2}/\left(f_{1}+f_{2}\right)$ in (29). The associated wavelength is $\lambda_{NL}=c/\left(f_{1}+f_{2}\right)$, equivalent to 10.7 cm for the GPS L1/L2 NL combination. NL code observations offer reduced noise levels compared with the original P1 and P2 codes.

#### 3.1.5. Melbourne–Wübbena (MW) Function

The MW function, denoted as $A_{MW}$, calculates the difference between the WL phase and NL code and can be expressed as

(31) $$A_{MW}=\Phi_{WL}-P_{NL}$$

This function possesses characteristics of both IF and GF combinations. It remains independent of geometrical models, orbits, clocks, or receiver positions, relying solely on the WL ambiguity and hardware biases. Consequently, the MW function plays a crucial role in PPP ambiguity resolution and cycle slip detection [73].

### 3.2. Standard PPP

The standard PPP model is constructed based on IF combinations of DF GNSS pseudorange and carrier-phase measurements, employing float-ambiguity resolution [63]. The IF observations of a GNSS satellite can be obtained using (1), (27), and (28)

(32) $$\begin{array}{cc} P_{IF}=\rho^{\prime } & +c\left(dt^{r}-dt^{s}\right)+b_{P_{IF}}^{r}-b_{P_{IF}}^{s}+T-v+PCV_{IF}^{s}+APC_{IF}^{r}+\epsilon_{P_{IF}}\\ \Phi_{IF}=\rho^{\prime } & +c\left(dt^{r}-dt^{s}\right)+b_{\Phi_{IF}}^{r}-b_{\Phi_{IF}}^{s}+T-v+PCV_{IF}^{s}+APC_{IF}^{r}+\lambda_{IF}N_{IF} \end{array}$$

(33) $$\begin{array}{ll} & +\frac{\lambda_{w_{IF}}}{2\pi }\phi_{w}+\epsilon_{\Phi_{IF}} \end{array}$$

These equations demonstrate that IF combinations maintain the values of frequency-independent quantities, such as geometric range and tropospheric delay, while altering the frequency-dependent terms. The IF wavelength $\lambda_{IF}$ of two combined signals with frequencies $f_{A}$ and $f_{B}$ can be calculated using [74]

(34) $$\lambda_{IF}=\frac{\lambda_{A}\lambda_{B}}{i_{A}\lambda_{B}+i_{B}\lambda_{A}}$$

where $i_{A}$ and $i_{B}$ are two integers satisfying $i_{A}/i_{B}=-f_{A}/f_{B}$. For example, for the GPS L1 and L2 frequencies, $i_{A}=77$ and $i_{B}=-60$ result in $\lambda_{IF}\approx 6.3$ mm. For GLONASS, $i_{A}=9$ and $i_{B}=-7$ result in $\lambda_{IF}\approx 5.3$ cm for channel 0 (the central frequency of GLONASS FDMA channels). On the other hand, the wavelength of the phase wind-up term $\lambda_{w_{IF}}$ is directly computed as

(35) $$\lambda_{w_{IF}}=\alpha_{IF}\lambda_{A}+\beta_{IF}\lambda_{B}=\frac{c}{f_{A}+f_{B}}$$

The satellite clock corrections, derived from precise or broadcast ephemeris, are generated relative to a reference GNSS signal. For example, the clock reference is the IF P1/P2 code observations in GPS and GLONASS. The satellite code biases $b_{P_{IF}}^{s}$ for this combination are incorporated into these corrections. In the standard PPP model, the IF code satellite clock correction $dt_{P_{IF}}^{s}$ can be applied directly to both code and carrier-phase observations as long as integer ambiguity resolution is not required. The same concept can be applied to the receiver clock term, where the code clock term $dt_{P_{IF}}^{r}$ is employed. The float-ambiguity term $A_{IF}$ absorbs the difference between phase and code biases. Thus, for GPS, the IF observations can be rewritten as

(36) $$\begin{array}{cc} P_{IF}=\rho^{\prime } & +c\left(dt_{P_{IF}}^{r}-dt_{P_{IF}}^{s}\right)+T-v+PCV_{IF}^{s}+APC_{IF}^{r}+\epsilon_{P_{IF}} \end{array}$$

(37) $$\begin{array}{cc} \Phi_{IF}=\rho^{\prime } & +c\left(dt_{P_{IF}}^{r}-dt_{P_{IF}}^{s}\right)+T-v+PCV_{IF}^{s}+APC_{IF}^{r}+\lambda_{IF}A_{IF}+\frac{\lambda_{w_{IF}}}{2\pi }\phi_{w}+\epsilon_{\Phi_{IF}} \end{array}$$

where

(38) $$A_{IF}=N_{IF}+\frac{\left(b_{\Phi_{IF}}^{r}-b_{P_{IF}}^{r}\right)-\left(b_{\Phi_{IF}}^{s}-b_{P_{IF}}^{s}\right)}{\lambda_{IF}}$$

Finally, the PPP observations are corrected using the error models in Section 2 and the network precise corrections. The correction is performed as follows:

(39) $$\begin{array}{cc} P_{IF_{corr}} & =P_{IF}+c\left(dt_{P_{IF}}^{s}\right)-M_{h}Z_{h}+v-PCV_{IF}^{s}-APC_{IF}^{r}\\ & =\rho +cdt_{P_{IF}}^{r}+M_{w}Z_{w}+\epsilon_{P_{IF}} \end{array}$$

(40) $$\begin{array}{cc} \Phi_{IF_{corr}} & =\Phi_{IF}+c\left(dt_{P_{IF}}^{s}\right)-M_{h}Z_{h}+v-PCV_{IF}^{s}-APC_{IF}^{r}-\frac{\lambda_{w_{IF}}}{2\pi }\phi_{w}\\ & =\rho +cdt_{P_{IF}}^{r}+M_{w}Z_{w}+\lambda_{IF}A_{IF}+\epsilon_{\Phi_{IF}} \end{array}$$

The system of equations in (39) and (40) is used to estimate the PPP positioning solution. The unknowns include three position states, the receiver clock error, the wet tropospheric delay, and float ambiguities (one per satellite).

For other GNSSs, the ISB term has to be added separately to the previous equations because it cannot be lumped into the receiver clock. An example of a Multi-GNSS is GPS/GLONASS PPP, which is employed in the experimental results in Section 4. Using (39) and (40), the GPS/GLONASS model can be written as

(41) $$\begin{array}{cc} P_{IF_{corr}}^{G} & =\rho +cdt_{P_{IF}}^{r}+M_{w}Z_{w}+\epsilon_{P_{IF}}^{G} \end{array}$$

(42) $$\begin{array}{cc} \Phi_{IF_{corr}}^{G} & =\rho +cdt_{P_{IF}}^{r}+M_{w}Z_{w}+\lambda_{IF}^{G}A_{IF}+\epsilon_{\Phi_{IF}}^{G} \end{array}$$

(43) $$\begin{array}{cc} P_{IF_{corr}}^{R} & =\rho +cdt_{P_{IF}}^{r}+ISB_{G-R}+M_{w}Z_{w}+\epsilon_{P_{IF}}^{R} \end{array}$$

(44) $$\begin{array}{cc} \Phi_{IF_{corr}}^{R} & =\rho +cdt_{P_{IF}}^{r}+ISB_{G-R}+M_{w}Z_{w}+\lambda_{IF}^{R}A_{IF}+\epsilon_{\Phi_{IF}}^{R} \end{array}$$

where the subscripts *G* and *R* denote GPS and GLONASS, respectively.

GLONASS observations introduce an additional unknown, the ISB between GLONASS and GPS, in meters. It is also worth mentioning that the IF wavelength for GLONASS can be different for each satellite due to the employment of the FDMA technique in GLONASS legacy signals. The same Multi-GNSS model can be applied to Galileo, Beidou, and other constellations.

### 3.3. Ambiguity-Fixed PPP

While dealing with float ambiguities is more straightforward, it has been well-established that resolving integer ambiguities in PPP can significantly enhance convergence time and solution accuracy, particularly along the longitudinal axis [75]. The standard PPP model combines residual biases with the ambiguity term, as indicated in (38). These biases are known as the Uncalibrated Phase Delays (UPDs) or Fractional Cycle Biases (FCBs). For the sake of simplicity, we focus on GPS ambiguity fixing; nevertheless, the general principles apply to other constellations.

To retrieve the integer nature of the ambiguity, UPDs must be estimated separately from the ambiguity term. However, this is not feasible within the traditional PPP framework. To illustrate this, let us simplify the standard PPP model by introducing Single Differencing (SD) between satellites. The SD operator, represented as Δ, subtracts the observations of a reference satellite from corresponding observations of the other satellites, effectively eliminating all receiver biases. Assuming all necessary corrections are applied except the satellite clock error, SD PPP observations can be expressed as

(45) $$\begin{array}{cc} \Delta P_{IF} & =\Delta \rho -c\Delta dt_{P_{IF}}^{s}+\Delta M_{w}Z_{w}+\Delta \epsilon_{P_{IF}} \end{array}$$

(46) $$\begin{array}{cc} \Delta \Phi_{IF} & =\Delta \rho -c\Delta dt_{P_{IF}}^{s}+\Delta M_{w}Z_{w}+\lambda_{IF}\Delta N_{IF}+\left(\Delta b_{\Phi_{IF}}^{s}-\Delta b_{P_{IF}}^{s}\right)+\Delta \epsilon_{\Phi_{IF}} \end{array}$$

Even with SD, the system remains unsolvable, as the number of unknowns still exceeds the number of observations. Another challenge arises from the fact that the IF ambiguity for GPS has a short wavelength of $\lambda_{IF}=6.3$ mm, making it comparable to the observation noise. To address this, the IF ambiguity is often decomposed into two components as follows [73]:

(47) $$N_{IF}=17N_{1}+60\left(N_{1}-N_{2}\right)=17N_{1}+60N_{WL}$$

where $N_{1}$ and $N_{2}$ represent the ambiguity of the phase observations at the L1 and L2 GPS frequencies, respectively.

To overcome the rank deficiency issue, the PPP network model must be adjusted to estimate additional corrections that users can receive to resolve the integer ambiguities [76]. Various models, such as the Decoupled Clock (DC) model [77], the Integer Recovery Clock (IRC) model [78], and the UPD/FCB model [68], have been introduced for this purpose. All these models introduce a third observation to the PPP model—the MW function. The MW function with single differencing can be expressed as

(48) $$\Delta A_{MW}=\lambda_{WL}\Delta N_{WL}-\Delta b_{A_{MW}}^{s}+\Delta \epsilon_{A_{MW}}$$

where $\Delta b_{A_{MW}}^{s}$ and $\Delta \epsilon_{A_{MW}}$ denote the FCB and noise for $\Delta A_{MW}$, respectively. The MW FCB, also known as WL FCB, remains stable over time and can be considered a constant. Nevertheless, smoothing $\Delta A_{MW}$ still requires around 10 min, after which $\Delta b_{A_{MW}}^{s}$ can be estimated as the fractional component of $\Delta A_{MW}$ [68].

CNES, an IGS analysis center, was the first to provide corrections to enable PPP ambiguity fixing, employing the IRC model [79]. Later, other IGS centers and academic organizations also started to provide phase bias products [80]. The IRC model is similar to the DC model but has different parametrization [81]. Both models involve different satellite clock terms for code and phase observations as follows:

(49) $$\begin{array}{cc} \Delta P_{IF} & =\Delta \rho -c\Delta {\mathrm{dt}}_{P_{\mathrm{IF}}}^{s}+\Delta M_{w}Z_{w}+\Delta \epsilon_{P_{IF}} \end{array}$$

(50) $$\begin{array}{cc} \Delta \Phi_{IF} & =\Delta \rho -c\Delta {\mathrm{dt}}_{\Phi_{\mathrm{IF}}}^{s}+\Delta M_{w}Z_{w}+\lambda_{IF}\left(17\Delta N_{1}+60\Delta N_{WL}\right)+\Delta \epsilon_{\Phi_{IF}} \end{array}$$

(51) $$\begin{array}{cc} \Delta A_{MW} & =\lambda_{WL}\Delta N_{WL}-\Delta b_{A_{\mathrm{MW}}}^{s}+\Delta \epsilon_{A_{MW}} \end{array}$$

The IRC model in the above equations estimates and sends to the users three correction parameters, indicated in bold, namely the satellite code clock, satellite phase clock, and WL biases. The satellite phase biases in (50) are absorbed in the phase clock term. At the user’s end, after applying the received correction $\Delta b_{A_{\mathrm{MW}}}^{s}$ , $N_{WL}$ is fixed by smoothing the $\Delta A_{MW}$ observation to get rid of the noise term $\Delta \epsilon_{A_{MW}}$. Subsequently, $N_{WL}$ is substituted in (50) to resolve the $N_{1}$ ambiguity. Although $N_{1}$ is the L1 phase ambiguity, it is usually referred to as the NL ambiguity. The phase biases are called the NL FCBs, because $N_{1}$ is multiplied by $17\lambda_{IF}$, equaling $\lambda_{NL}$. The NL biases tend to vary more rapidly than their WL counterparts and, therefore, can be modeled as piecewise constants [68].

Even with corrections provided directly by the network, a substantial initialization period is still required to smooth the measurements and allow changes in satellite geometry for resolving the IAs [82]. Fixing the IA can be accomplished by simple rounding, bootstrapping, or more advanced integer least-squares techniques like the Least-squares AMBiguity Decorrelation Adjustment (LAMBDA) method [83,84].

### 3.4. PPP-RTK

GNSS precise centimeter-level positioning can be achieved by RTK or PPP. RTK has the advantage of short convergence time, e.g., a few seconds. Nevertheless, RTK depends on the communication between the rover receiver and one or more reference stations, introducing baseline and bandwidth limitations. On the other hand, PPP enables the absolute positioning of a single receiver, but the convergence time of the ambiguity-fixed PPP, or PPP-AR, exceeds 20 min [77,78].

The term “PPP-RTK” was introduced in [85] to reduce the gap between PPP and RTK and enable centimeter-level accuracy in a few seconds with a single rover receiver. Although “PPP-AR” and “PPP-RTK” are used interchangeably in many research papers to denote the PPP with ambiguity resolution, in this article, we adopt the convention that PPP-RTK is not the same as PPP-AR [69,85].

As described in Section 3.3, PPP-AR is the ambiguity-fixed PPP employing the standard IF observations. In real-time PPP-AR, the receiver obtains precise corrections for the satellites’ orbits, clocks, and biases. The IF combinations mostly eliminate the ionospheric delay; nonetheless, they contain more noise than their corresponding uncombined observations, as illustrated in Section 2.1. Therefore, PPP-AR requires a long convergence time.

In contrast, in PPP-RTK, the atmospheric errors are estimated on the network side and sent to the users along with the other precise corrections to enable fast ambiguity resolution. Furthermore, the PPP-RTK model can rely on a global network of reference stations on the server side or a regional network similar to RTK for a more accurate estimation. While RTK adopts the Observable Space Representation (OSR), PPP-RTK, similar to real-time PPP-AR, employs the State-Space Representation (SSR), reducing the communication bandwidth and allowing more users to be served.

In summary, PPP-RTK is a state-of-the-art precise positioning model that combines the advantages of both PPP and RTK. More details on PPP-RTK can be found in [69,76,85].

## 4. Practical Considerations and Experimental Results

In this section, the implementation and error modeling of the standard DF-PPP is demonstrated using static and kinematic data. In both scenarios, only observations from GPS and GLONASS systems were available from the GNSS receivers; therefore, only these two constellations were utilized in the implemented PPP solution.

Essential correctional components—namely the final satellite orbit, clock, and DCB corrections—were sourced from the CODE analysis center, part of the IGS MGEX project. The satellite orbit corrections were provided every 5 min, whereas the satellite clock corrections every 30 s.

The Extended Kalman Filter (EKF) was employed as the navigation filter for static and kinematic datasets. However, some practical aspects must be considered when processing static versus kinematic data. The PPP processing was performed with an in-house MATLAB code. It is worth mentioning that the results in this section are examples of what can be expected of the standard DF-PPP performance in static and kinematic modes. The performance metrics cannot be statistically generalized, as this requires longer and more diverse data, which is beyond the scope of this article.

### 4.1. Static Results

Static PPP offers distinct advantages in specific applications, notably surveying and mapping. Conventionally, static PPP algorithms undergo evaluation using data from IGS stations. In this work, we utilized the daily GNSS observations of IGS stations, available in RINEX format, and a 30-second sampling rate through the IGS data center [86]. The YELL station was chosen randomly for static PPP testing. The station reference position is obtained from the SINEX (Solution INdependent EXchange) file, one of the IGS products (it comes with the extension (*.snx)).

Our measurements were based on pseudorange and carrier-phase observations, with measurement standard deviation (STD) values determined inversely proportional to the squared sine of satellite elevation angles. An elevation mask of $7^{\circ }$ was also applied to filter out low-elevation satellites. Since carrier-phase measurements are much more precise than pseudoranges, the carrier-phase STD was reduced to be (1/100) of the code STD. To account for variations in signal-to-noise ratios and signal structures between GPS and GLONASS, STD values for GLONASS code observations were scaled by a factor of 3, and phase observations were scaled by a factor of 1.5, as detailed in [87].

As PPP standalone positioning lacks a deterministic system model, we relied on the stochastic characteristics of the states to describe uncertainties in the system model (Q matrix). In our model, the three position parameters were considered random constants. The receiver clock bias was modeled as white noise, whereas the ISB between GLONASS and GPS, along with the zenith wet delay, were modeled as random walk processes. Float ambiguities were treated as random constants, with initial parameters obtained from a Least Squares (LS) SPP solution.

**Figure 4:**
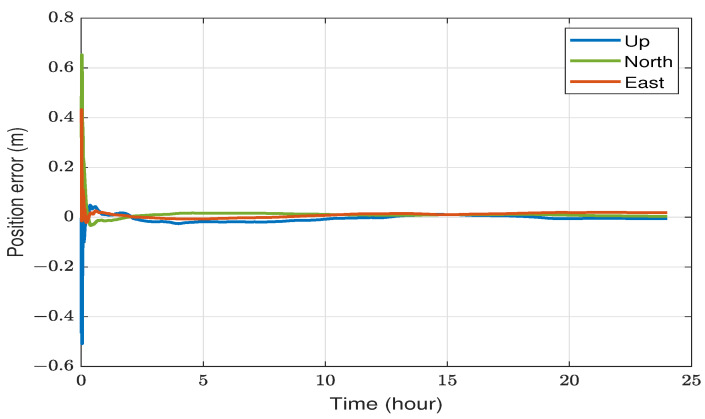
An illustration of static DF-PPP positioning utilizing data from the YELL station on 20 July 2023.

Figure 4 depicts the 3D position errors for the YELL station in Yellowknife, Canada, observed over a 24-hour period on 20 July 2023. The number of visible GPS/GLONASS satellites fluctuated between 15 and 22 during the day. The position root mean square errors (RMSE) measured 1.2 cm, 1.1 cm, and 1.3 cm for the east, north, and up directions, respectively. These errors were calculated after the initial 20 min to exclude the impact of initial convergence. All three errors converged to fewer than 10 cm after approximately 10 min and fewer than 2 cm after about 45 min.

In some applications with a mix of kinematic and static dynamics, the assumption that the receiver is static is no longer valid. Therefore, the data have to be processed in a kinematic mode, which means the receiver position can change every epoch and cannot be modeled as a random constant. For instance, in land vehicle navigation, positions can be modeled as random walk processes with substantial uncertainty, e.g., (100 $m/\sqrt{s}$). Also, the position can be initialized every epoch from an LS SPP. Motion constraints, if there are any, can also be applied.

**Figure 5:**
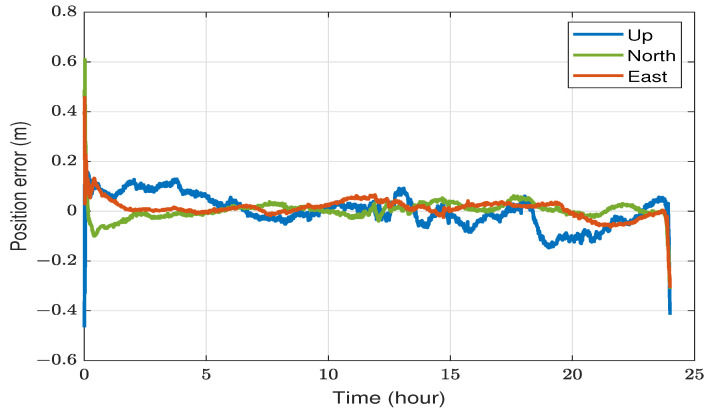
An illustration of DF-PPP of static data in a kinematic mode utilizing data from YELL station on 20 July 2023.

We processed the same data from the YELL station in kinematic mode, disregarding the static assumption, to illustrate the different impacts of static and kinematic positioning on static data. As shown in Figure 5, position errors exhibit less smoothness than the static case. Furthermore, the RMSE errors are relatively higher, measuring 3.1 cm, 2.6 cm, and 6.3 cm for the east, north, and up directions, respectively. These values are calculated excluding the first 20 min and the last hour. The spikes in errors at the end of the day result from the interpolation of precise satellite corrections using edge points, causing a lack of centered interpolation points. In contrast, such interpolation at edge points had a lesser effect on the static case because the assumption of constant receiver location prevented abrupt position changes. While the east and north position errors dropped below 10 cm after around 30 min, the up direction took a few hours and was not as stable.

### 4.2. Kinematic Results

A road test was conducted in Calgary, Canada, spanning approximately 50 min, predominantly on highways. GNSS data for the test were captured using the NovAtel SPAN OEM6 receiver, a dual-frequency GNSS receiver incorporated within the NovAtel SPAN ProPak6 system.

**Figure 6:**
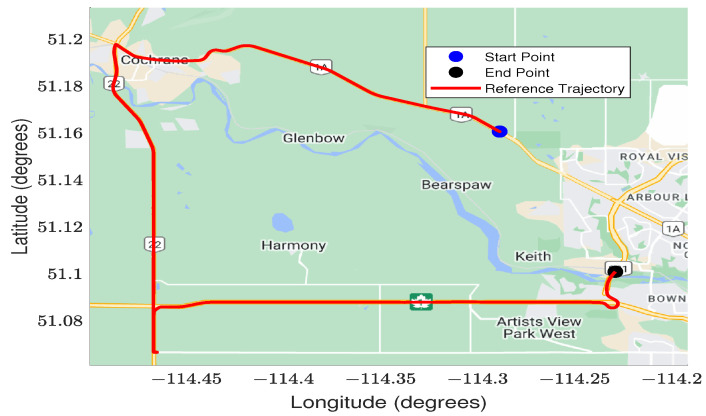
The kinematic test trajectory , conducted in Calgary, Alberta, Canada.

A reference solution was derived from the SPAN unit by leveraging its tactical-grade IMU-KVH. The data were postprocessed in a tightly coupled DGNSS/INS mode utilizing NovAtel’s Waypoint Inertial Explorer Software. The base station data were acquired from the UCAL IGS station with a baseline of fewer than 30 km. Figure 6 provides a visual representation of the test trajectory.

**Figure 7:**
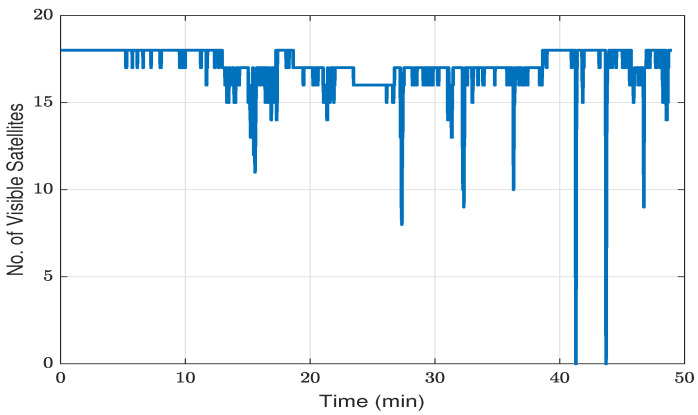
Number of visible GPS/GLONASS satellites observed during the kinematic test.

The visible GPS/GLONASS satellite fluctuations during the test are visually presented in Figure 7. In the initial 40 min, characterized primarily by open-sky conditions, the count of visible satellites fluctuated between 8 and 18. However, a notable drop to zero satellites was observed as the vehicle transitioned under two sequential overpass bridges.

**Figure 8:**
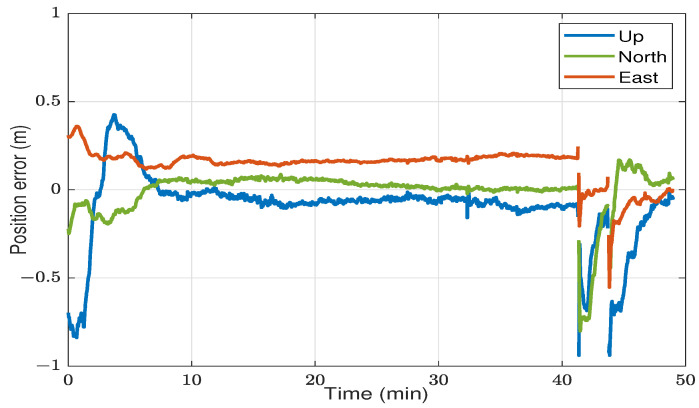
Position errors in the kinematic test.

Figure 8 shows the 3D position errors of the standard DF-PPP solution versus time. The solution converged to a less than 20 cm error level after 5.5 min. The solution remained within the same accuracy level until the first overpass. When the number of satellites dropped to zero, there were no observations, and the navigation filter had to be reset so the ambiguities could be re-estimated. Then, the solution started to reconverge again until the second overpass. These data exemplify the PPP initial convergence, stable open-sky solution, and reconvergence in GNSS-challenging environments. During the stable period after the initial convergence, from the 10th minute to the 40th minute, the RMSE measured 17 cm, 4 cm, and 7 cm for the east, north, and up directions, respectively.

## 5. PPP Challenges and Solutions

The experimental results demonstrate the advantage of PPP as a global precise GNSS solution with high accuracy. However, achieving the full potential of PPP requires addressing several challenges. A predominant challenge is the protracted convergence time, often exceeding 20 min in standard DF-PPP, attributed to the time necessary for carrier-phase ambiguity resolution. Multiple factors influence this convergence duration, including the number of visible satellites, satellite geometry, correction product quality, receiver multipath environments, and prevailing atmospheric conditions. Extensive research is underway to expedite PPP convergence, aligning it with the demands of real-time applications. Recent studies have indicated that employing triple-frequency, multiconstellation, and adopting ionospheric corrections instead of IF combinations could potentially shorten PPP convergence time [72,88,89]. Moreover, fixing the integer ambiguities can also reduce the convergence time to some extent compared with the float solution; nonetheless, it necessitates supplementary corrections and more complicated algorithms. A state-of-the-art method that combines the advantages of PPP and RTK and allows faster ambiguity resolution is the PPP-RTK.

Another significant challenge for PPP is the performance deterioration or potential inability to provide a solution in challenging GNSS environments like underpasses, within tunnels, or amid towering buildings. Although this limitation is not exclusive to PPP and pervades all GNSS positioning modes, its ramifications are magnified in PPP due to the requisite reconvergence time to reclaim the original precision. This particular constraint is of paramount concern in scenarios like land vehicular navigation. Therefore, the fusion of PPP with other navigation systems is employed to ensure the continuity of the navigation solution and reduce the PPP reconvergence time. Two examples of this fusion are PPP/INS integration [9,11,90] and PPP/INS/Vision/LIDAR integration [91] for navigation in GNSS-challenging environments.

## Data Availability

GNSS observations and precise correction products are open-access, with free registration, through the International GNSS Service (IGS) at https://igs.org/, accessed on 6 August 2023.
